# Métastase vésicale d'un adénocarcinome gastrique en bague à chaton

**DOI:** 10.11604/pamj.2014.19.206.4966

**Published:** 2014-10-27

**Authors:** Youssef Seddik, Ouissam Al Jarroudi, Sami Aziz Brahmi, Said Afqir

**Affiliations:** 1 Service d'Oncologie Médicale, CHU Mohammed VI, Université Mohammed Premier, Oujda, Maroc

**Keywords:** Métastase vésicale, adénocarcinome gastrique, cellules en bague à chaton, Bladder metastasis, gastric adenocarcinoma, signet ring cells

## Abstract

Bien que le cancer primitif de la vessie représente le deuxième cancer urologique après le cancer de la prostate, les métastases vésicales sont rares et le primitif gastrique reste exceptionnel. On rapporte le cas d'un patient âgé de 46 ans, avec des épigatsralgies chroniques, qui présentait une hématurie totale avec des signes irritatifs urinaires. La cystoscopie a montré une vessie inflammatoire sans lésion tumorale décelable. La biopsie vésicale avec une étude anatomopathologique a été en faveur d'un adénocarcinome peu différencié à cellules indépendantes. Le scanner thoraco-abdomino-pelvien a montré un épaississement vésical et gastrique. La fibroscopie oeso-gastroduodénale a montré une tumeur bourgeonnante ulcérée fundique qui a été biopsiée. L’étude anatomo-pathologique a montré un adénocarcinome gastrique à cellules indépendantes mucipares. Le patient a eu 6 cures de chimiothérapie palliative de première ligne type EOX à base d'Epirubicine, Oxaliplatine, et Capécitabine, avec une légère régression tumorale puis une chimiothérapie de maintenance à base de Capécitabine.

## Introduction

Au Maroc, Le cancer de la vessie occupe le 3ème rang en termes de fréquence chez les hommes avec une incidence 11 fois plus élevée que chez les femmes. Le carcinome urothélial est le type histologique le plus fréquent (82%) [1]. Les métastases vésicales sont rares, elles représentent seulement 2,3% de toutes les tumeurs malignes de la vessie. L'estomac représente 4,3% de l'ensemble des localisations qui donne des métastases vésicales [2].

## Patient et observation

**Figure 1:**
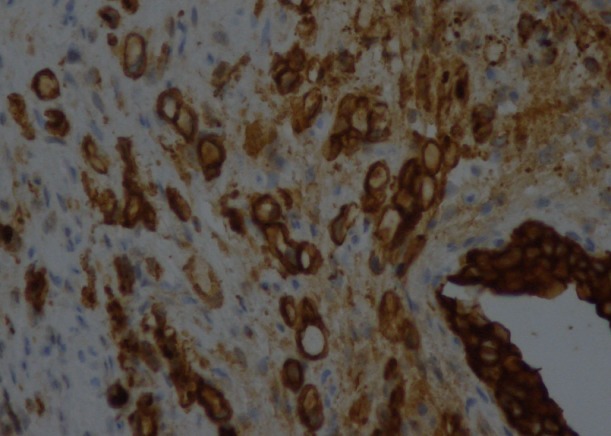
La muqueuse vésicale est infiltrée par des cellules tumorales dotées de gros noyaux hyper chromatiques refoulés en périphérie par un cytoplasme abondant leur donnant un aspect en « bague à chaton »

**Figure 2:**
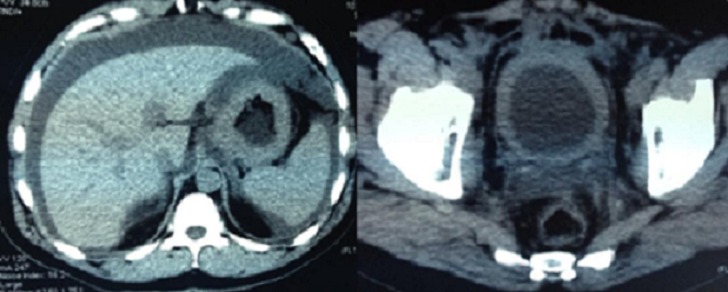
Le scanner thoraco-abdomino-pélvien montre un bourgeon tumoral intraluminal gastrique, un épaississement de la paroi vésicale et gastrique, des nodules de carcinose péritonéale, des adénopathies intra-abdominales, et une ascite de grande abondance

**Figure 3:**
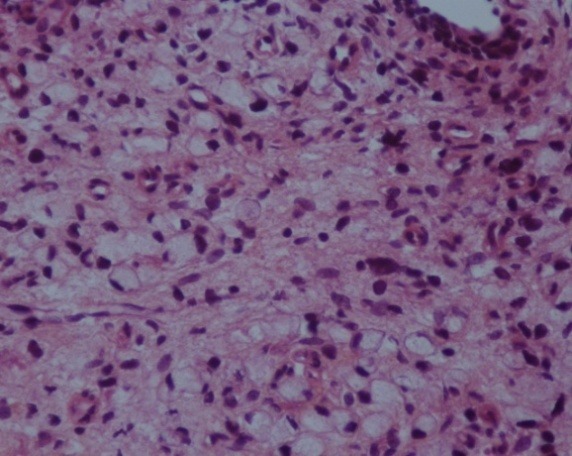
La muqueuse gastrique est le siège d'une prolifération massive de cellules carcinomateuses d'aspect en « bague à chaton » isolées ou agencées en plages compactes

**Figure 4:**
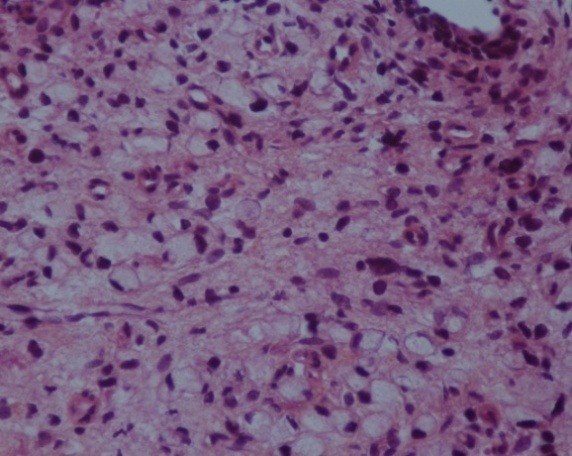
La confrontation immunohistochimique montre le marquage positif aux Ac anti CK20 et négatif aux Ac anti CK7

Il s'agit d'un patient âgé de 46 ans. Il a la notion d’épigastralgies chroniques qui n'ont jamais été explorées. Il a présenté une symptomatologie urinaire faite d'hématurie totale avec des signes irritatifs urinaires type pollakiurie et dysurie, évoluant dans un contexte d'amaigrissement non chiffré. La cystoscopie a montré une vessie inflammatoire sans lésion tumorale décelable.la biopsie vésicale avec une étude anatomopathologique a été en faveur d'un adénocarcinome peu différencié à cellules indépendantes (Figure 1). Le scanner thoraco-abdomino-pelvien a montré un bourgeon tumoral intraluminal gastrique, un épaississement de la paroi vésicale et gastrique (Figure 2), des nodules de carcinose péritonéale, des adénopathies intra-abdominales, et une ascite de grande abondance. La fibroscopie oeso-gastro-duodénale a montré une tumeur bourgeonnante ulcérée au niveau fundique qui a été biopsiée. L’étude anatomo-pathologique a été en faveur d'un adénocarcinome gastrique à cellules indépendantes mucipares (Figure 3). La confrontation anatomopathologique et immunohistochimique entre la biopsie gastrique et vésicale a été en faveur d'un adénocarcinome primitif gastrique en bague à chaton métastatique au niveau de la vessie avec des anticorps anti AE1/AE3 et anti CK7 très positifs, et des anticorps anti CK20 et anti CDX2 négatifs (Figure 4). L'hercept-test a été négatif. Le marqueur tumoral ACE était normal, alors que le CA 19-9 a été très élevé: 35 x la valeur normale.

Le patient a eu 6 cures de chimiothérapie palliative de première ligne type EOX associant: Epirubicine = 50mg/m^2^, Oxaliplatine = 180 mg/m^2^ et Capécitabine = 625 mg/m^2^ en continu; une cure chaque 21 jours. Avec une amélioration clinique et une légère régression tumorale. Puis une chimiothérapie de maintenance à base de Capécitabine seule à la dose de 2500 mg/m^2^. Après 5 cures de mono chimiothérapie, le patient a présenté un syndrome d'hypertension intracrânienne, qui n'a pas été exploré vu que le malade est décédé le jour même.

## Discussion

Les métastases vésicales sont rares, elles représentent seulement 2,3% de toutes les tumeurs malignes de la vessie. On distingue deux types d'extension vers la vessie: Une extension par contigüité: à partir du colon, de la prostate, du rectum et du col utérin (par ordre décroissant); et une extension à distance: à partir du sein, du mélanome, ainsi que de l'estomac avec un pourcentage de 31,1% [3].

Dans notre cas, la rareté relative de l'adénocarcinome primitif de la vessie en plus de l'association d'un cancer de l'estomac, ont rendu difficile la détermination du primitif. Mostofi et al proposent qu'on peut retenir le diagnostic de l'origine vésicale de l'adénocarcinome si la muqueuse contient des formations polypoïdes, des nids de Brunn, une métaplasie glandulaire ou s'il y a des cellules transitionnelles associées [4]. Csilla András et al. ont retenu le diagnostic de métastase vésicale d'un adénocarcinome primitif gastrique devant des anticorps anti CK7, anti EMA, anti ACE, et anti CA19-9 positifs; et des anticorps anti CK20 négatifs [2]. L'adénocarcinome en bague à chaton est un type histologique extrêmement rare des cancers de la vessie avec un pourcentage de 0,24%. Une fois retrouvé, il doit faire évoquer une origine secondaire d'un primitif à distance à savoir d'origine digestive [3–5]. Dans notre cas, la présence des cellules en bague à chaton avec des anticorps anti AE1/AE3 et anti CK7 positifs, et des anticorps anti CK20 et anti CDX2 négatifs était suffisante pour retenir le diagnostic d'un primitif gastrique.

A notre connaissance, de très rares cas de cancer gastrique en bague à chaton métastatique au niveau de la vessie ont été rapportés dans la littérature. Ce sont le plus souvent des cas japonais. L'adénocarcinome en bague à chaton gastrique est une entité clinico-histopathologique particulière du cancer de l'estomac, redoutée par sa chimio-résistance et son mauvais pronostic [6]. A l'instar de l'adénocarcinome gastrique, le traitement est basé sur la chirurgie radicale encadrée par la CMT péri-opératoire ou suivie de la radio-chimiothérapie concomitante dans les stades localisés [7]. Alors qu'au stade métastatique, la chimiothérapie de 1ère ligne est basée sur l'Epirubicine, Cisplatine, 5 Fluoro-Uracile (5FU) [8] tout en ayant la possibilité de remplacer le Cisplatine par l'Oxaliplatine et le 5FU par la Capécitabine [9].

## Conclusion

L'adénocarcinome en bague à chaton de la vessie est un type histologique extrêmement rare qui nous pousse à rechercher le primitif dans le tractus digestif. Les métastases vésicales d'un primitif gastrique restent exceptionnelles.
